# Deciphering the role of claudins in lung cancer

**DOI:** 10.3389/fonc.2024.1435535

**Published:** 2024-09-19

**Authors:** Tarek Ziad Arabi, Wael Alkattan, Nadine Ashraf Osman, Belal Nedal Sabbah, Nader Ashraf, Abderrahman Ouban

**Affiliations:** College of Medicine, Alfaisal University, Riyadh, Saudi Arabia

**Keywords:** lung, cancer, claudins, treatment, diagnosis, pathogenesis

## Abstract

Lung cancer remains a major global health challenge, characterized by aggressive malignancy and poor prognostic outcomes. This review article focuses on the pivotal role of claudins, a family of tight junction proteins, in the pathophysiology of lung cancer. Claudins are integral to maintaining epithelial barrier function and cellular polarity, yet they are intricately involved in the progression and metastasis of lung cancer. The aberrant expression of claudins has been observed across various histological subtypes of lung cancer, indicating their potential as diagnostic and prognostic biomarkers. Specifically, claudins such as claudin-1, -2, -3, -4, and -7 exhibit diverse expression patterns that correlate with tumor aggressiveness, patient survival rates, and response to therapies. Inflammation and cytokine modulation significantly influence claudin expression, affecting tumor microenvironment dynamics and cancer progression. This review also highlights the therapeutic implications of targeting claudins, particularly in cases resistant to conventional treatments. Recent advances in this area suggest that claudin-modulating agents may enhance the efficacy of existing therapies and offer new avenues for targeted interventions. By integrating the latest research, this article aims to provide a comprehensive understanding of claudin’s roles in lung cancer and encourages further clinical trials to explore claudin-targeting therapies. This could pave the way for more effective management strategies, improving outcomes for lung cancer patients.

## 1 Introduction

Lung cancer is the leading cause of cancer incidence and deaths globally (1, 2). Recent trends indicate a decreasing incidence of lung cancer in many nations, attributed to a decline in smoking habits (3). The five-year relative survival rates for small cell lung carcinoma (SCLC) and non-SCLC (NSCLC) ranges from 3%–30% and 9%–65%, respectively, depending on the tumor’s stage (4). However, advances into lung cancer molecular biology and immune-based treatments are expected to improve these statistics in the future (5–7).

To understand the complex nature of lung pathologies, it is essential to explore its biological underpinnings, starting at the cellular level where the disease initiates and progresses (8). The lung’s barrier function, crucial for the free diffusion of solutes into airspaces, is facilitated by epithelial cells and tight junctions, comprising key proteins such as occludin, zona-occluden, desmosomes, and claudins (9–11). These structures not only regulate the permeability of the epithelial barrier but also control the passage through the paracellular space (12). Tight junctions are not only vital for barrier integrity, but also facilitate cellular proliferation and differentiation and maintain cellular polarity, an essential factor in cellular communication and signaling (13–17).

Claudins, a family of tetraspan transmembrane proteins, are integral to this barrier function for tight junction (18, 19). All claudins share a similar secondary structure, with significant variation observed in their extracellular domains and cytoplasmic scaffolding interactions, particularly among non-classic claudins (20). The differential expression of claudins across various histological types of lung tumors could be partially attributed to the originating cell type of the tumor (21). Claudins-1, -3, -4, -5, and -7 are predominantly expressed, with each showing distinct patterns of localization and function (22–24). For instance, claudin-18 is primarily found in alveolar epithelium while claudins-4 and -7 are more ubiquitously expressed throughout the respiratory epithelium, highlighting the diversity of claudin expression in different lung regions (23, 25, 26).

In lung cancer, claudins may play an essential role as disease markers, with their expression levels inversely correlating with tumor aggressiveness and patient prognosis (27). Claudins -1, -2, -3, -4, and -7 exhibit diverse expression patterns in lung carcinoma, with variations among different histological types compared to normal lung tissue and even being influenced by smoking habits (28–31). Emerging research also indicates a link between claudin expression and lung cancer metastasis, suggesting their involvement in tumor migration and invasion (32–34). Also, claudin expression has been linked to epithelial-mesenchymal transition in lung squamous cell carcinoma (LSCC) cells through the Tyk2/Stat1 (35) or Wnt/β-catenin signaling pathways (36).

Building upon previous comprehensive reviews which illuminated the role of claudins in head and neck (37), gastrointestinal (38), and genitourinary cancers (39), this paper extends the investigation into the realm of lung cancer. Exploring the intricate mechanisms behind the aberrant expression of claudins in lung cancer is essential, as it may lay the groundwork for identifying novel therapeutic targets in upcoming clinical trials. This paper aims to deliver a comprehensive review of the claudin expression patterns observed in lung cancer and their potential applications in monitoring and managing the disease. We will also delve into the molecular mechanisms leading to irregular claudin expression and discuss strategies for targeting these aberrations in treating lung cancer.

## 2 Claudins in non-small cell lung cancers

NSCLCs, which include lung adenocarcinomas (LUAD), LSCC, and large cell carcinomas, constitute nearly 85% of all histological lung cancer types (40). NSCLCs are more strongly linked to smoking, which is attributed to three-quarters of all lung cancers globally, than SCLCs (41). Despite advancements in NSCLC management, the unclear mechanisms of disease progression and need for a larger portfolio of targeted therapies leave a lot to be achieved in the future (40). Claudins have been studied extensively in NSCLCs to fill in the gap and guide novel prognostic and therapeutic strategies. In this section, we summarize the evidence describing the expression of claudins in NSCLCs, their disease-modifying effects, and potential prognostic implications (**Table 1**).

**Table 1:** Changes in the levels of expression of different claudins in lung cancers compared to normal tissue.

Malignancy	CLDN-1	CLDN-2	CLDN-3	CLDN-4	CLDN-5	CLDN-7	CLDN-18
LUAD	↓	↑	↑	↑	N/A	↓	N/A
LSCC	↑	N/A	↓	N/A	↓	↓	↓
SCLC	N/A	↓	↑	↑	N/A	↑	N/A

↑: increased expression. ↓: decreased expression.

### 2.1 Claudins in lung adenocarcinomas

**Figure 1:**
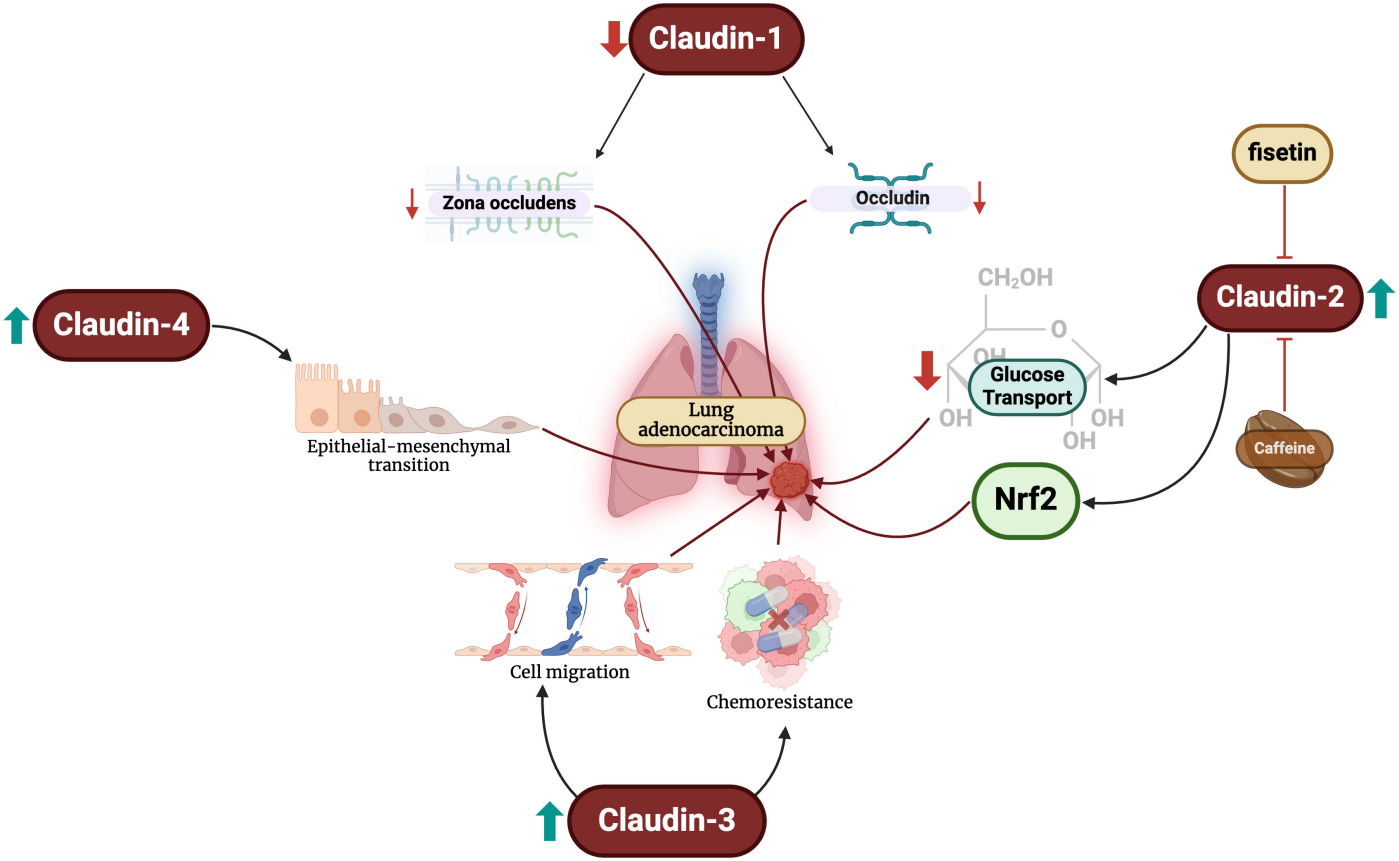
Changes in claudin levels promote the development of LUAD through different mechanisms. Firstly, reductions in claudin-1 levels lead to decreased expression of zona-occludens and occludin. Increased claudin-2 levels blunt glucose transport and activates Nrf2. Furthermore, claudin-3 overexpression promotes malignant cell migration and chemoresistance. Finally, claudin-4 exacerbates epithelial-mesenchymal transition, an important step in oncogenesis.

LUADs are the most common type of lung cancers globally, and the incidence continues to climb (42). Emerging evidence has demonstrated that aberrant expression of the claudin family mediates LUAD progression (**Figure 1**). LUADs have reduced claudin-1 messenger ribonucleic acid (mRNA) expression and an absence of the protein (43). When present, claudin-1 can be primarily seen along the tight junctions of LUAD cells, accompanying zona occludins-1 (44). The protein suppresses LUAD cell migration, invasion, and metastasis (45). Claudin-1 also modulates the genetic profile of LUAD cancer cells by promoting the expression of metastasis suppressors and blunting that of metastasis promotors (45). Some studies have revealed that low expression of the protein is independently associated with poor overall survival in these patients (45, 46), while others have been unable to demonstrate an association (47, 48). On the contrary, Sun et al. found that claudin-1 overexpression is associated with poor survival (49). The reasons for the conflicting data are unclear; nevertheless, claudin-1 appears to play important mechanistic and prognostic roles in LUAD patients.

Claudin-2 has also been extensively study in LUAD. Claudin-2 is highly expressed in LUAD compared to normal lung cells (32, 50, 51). Additionally, claudin-2 protein expression in LUAD is significantly greater than that of SCLCs, elucidating its potential diagnostic value (52). Arai et al. demonstrated that claudin-2 promotes LUAD cell proliferation (50). Claudin-2 promotes chemoresistance to doxorubicin of LUAD cells by inhibiting glucose transport and activating the nuclear factor erythroid 2-related factor 2 (Nrf2) signaling pathways (53). Targeting these pathways, caffeine administration has been shown to enhance lysosomal degradation of claudin-2 and, subsequently, reducing Nrf2 activity and enhancing chemosensitivity (54). Similar effects are also exhibited by fisetin – a flavonoid – and kaempferide through Akt pathway inhibition (55, 56).

Claudin-3 is exhibited in normal bronchial cells in a circular manner at the cellular membrane, but not in pneumocytes (48). The diagnostic utility of claudin-3 appears to be limited in LUADs. Moldvay et al. found increased levels of protein expression in LUADs compared to LSCC (52), while Jung et al. found no difference between the two entities (48). Claudin-3 expression has been linked to poor survival and increased recurrence and/or metastasis in LUAD patients, but not tumor stage or size (57). Forced overexpression of claudin-3 enhances LUAD cell migration, proliferation, and chemoresistance, and its knockout blunts these effects (57).

Claudin-4 is focally expressed in normal pneumocytes and more strongly expressed in the cylindrical cells of the bronchial mucosa (48). Claudin-4 expression is significantly upregulated in LUADs compared to normal and premalignant samples (52, 58, 59) and significantly greater than that of LSCCs (48, 52). Claudin-4 can also be used to differentiate between LUAD and malignant pleural mesotheliomas (60), and specifically epithelioid mesothelioma with a sensitivity and specificity of 97% (61). However, the protein is not associated with any staging or survival parameters in LUAD patients (48). To our knowledge, there have been no studies examining the mechanistic role of claudin-4 in LUAD development. However, changes in claudin-4 expression have been shown to promote epithelial-mesenchymal transition, which is a crucial predisposing factor to the neoplastic process (62). It is crucial for future studies to elucidate the role of claudin-4 in LUADs.

Claudin-7 proteins are strongly expressed in normal bronchial cells (52). In LUADs, mRNA expression does not differ from that in normal tissues (52), but protein levels are significantly reduced (63). Similarly, mRNA levels in LUADs are similar to LSCCs; however, immunohistochemistry has shown elevated levels in LUAD samples (52).

### 2.2 Claudins in lung squamous cell carcinomas

Following LUAC, LSCCs constitute the second most diagnosed form of NSCLCs (64), resulting in nearly a third of lung cancer in men and a fifth of lung cancer in women (65). Besides their utility in differentiating between LUAC and LSCC, as discussed previously (48, 52), alterations in LSCC compared to normal tissue and prognostic implications have also been reported. Overexpression of claudin-1 mRNA is found in up to 80% of LSCC samples (66, 67). Further solidifying its role, forcibly silencing claudin-1 expression blunts LSCC cell line proliferation and invasiveness (67). However, there has been no association found between its expression and the prognosis of LSCC patients (47).

Reduced claudin-3 levels have been linked to poor overall survival in LSCC patients, due to its promotion of epithelial-mesenchymal transition (68). Che et al. also demonstrated that claudin-7 is downregulated in LSCCs, and its downregulation is linked with poor differentiation and lymphatic metastasis (63). Blunted levels of claudins -5, -7, and -18 promote proliferation of LSCC cells due to phosphorylation and nuclear translocation of Akt and subsequent activation of the G_1_/S transition in the cell life cycle (69). Finally, claudin-12 has been shown to drive epithelial-mesenchymal transition through activation of the Tyk2/Stat1 signaling (70). Conclusively, claudins are drivers of LSCC pathogenesis; however, further studies are needed to elaborate on their mechanistic roles and prognostic potential.

## 3 Claudins in small cell lung carcinoma

SCLC is a highly aggressive form of lung cancer, responsible for a significant number of cancer-related deaths worldwide (71, 72). The malignancy of SCLC is characterized by its aggressive biological features, especially its high propensity for metastasis, contributing to its poor prognosis (73, 74).

In the context of claudins, the expression of these proteins in SCLC reveals critical insights into its pathology. Molday et al. observed SCLCs showing strong immunopositivity with claudins-3, -4, and -7, while being less strong for claudin-1 and being entirely negative for claudin-2 (52). However, Sormunen et al. displayed that SCLC exhibit a higher expression of claudin-2 than adenocarcinomas (21), contradicting with Molday et al. (52). Additionally, claudins-3 and -4 mRNA expression in SCLC are found to be 16 times and 3–4 times, respectively, higher than in normal lung tissue (52). Furthermore, in comparison to carcinoid tissue, claudin-3 and -4 mRNA expression in SCLC was found to be 13 times and 3 times higher, respectively (52). Nonetheless, LSCC has 15 times higher claudin-3 mRNA expression compared to SCLC (52). These findings highlight the distinct claudin expression patterns in SCLC, suggesting their potential role in the disease’s aggressive nature.

Expanding upon these observations, further research into claudin expression in SCLC may provide valuable perspectives on key aspects of the disease’s progression and intervention. For instance, Mao et al. identified exosomal miR-375-3p to break the vascular barrier by inhibiting claudin-1, promoting SCLC metastasis (33). In addition, Spi-B–mediated silencing of claudin-2 promotes early dissemination of both SCLC and NSCLC (75). Recently, the identification of differentially expressed genes in SCLC showed claudin-18, among others, significantly correlated to immune infiltration in the tumor microenvironment (76). Moreover, a study identified junctional adhesion molecule 3 as a potential therapeutic target, further reinforcing the importance of exploring these molecules in the development of new treatments for SCLC (77). These insights pave the way for future research, emphasizing the need for a deeper understanding of molecular mechanisms in SCLC to enhance therapeutic strategies and improve patient outcomes.

## 4 Claudins in pleural mesothelioma

Malignant pleural mesothelioma (MPM) affects 2,000 to 3,000 people annually in the United States and is rising worldwide, with over 5,000 new cases yearly in Western Europe and a peak incidence expected in Japan by 2025 (78–80). MPM, which is more common in men (5:1 male-to-female ratio) and increases with age, is primarily related to occupational asbestos exposure, though familial cases and other factors, such as previous radiation and simian virus-40, have also been identified (78, 81–85). Despite its histologic diversity (epithelioid, biphasic, and sarcomatoid), there are no approved early detection methods, but serum mesothelin-related peptide and osteopontin show promise in diagnosis (86, 87). To date, treatment remains challenging due to the disease’s complexity and limited patient numbers for studies, resulting in an alarmingly low median survival of 9 to 17 months (78). However, recent developments suggest potential advancements in MPM treatment (88, 89).

Studies have shown distinct expression patterns of various claudin subtypes in mesothelioma tissues, with these patterns often being linked to the specific type, severity, and expected outcome of the tumor. In comparison to normal mesothelial tissues, mesothelioma tissues frequently display different claudin expression profiles, hinting at their possible involvement in the development or progression of the cancer. In their study, Soini et al. observed that 40%, 80%, 18%, 23%, 14%, and 43% of mesothelioma cases expressed claudins -1, -2, -3, -4, -5, and -7, respectively (90). Notably, the presence of claudins -1, -3, -4, -5, and -7 was markedly lower in mesothelioma compared to metastatic adenocarcinoma, except for claudin 2, which showed no significant difference. Furthermore, an inverse relationship was found between the presence of claudins -1, -3, -4, -5, and -7 and calretinin positivity. In terms of mesothelioma subtypes, sarcomatoid and biphasic forms showed less positivity for these claudins compared to the purely epithelioid form. However, the study found no link between claudin expression and the survival rates of patients with malignant mesotheliomas (90). Interestingly, Nakashima et al. (91) found that claudin-5 was not expressed in mesothelioma, presenting a stark contrast to the findings of Soini et al. (90), who reported claudin-5 expression in 14% of mesothelioma cases. This contradiction between the studies could potentially be attributed to a bias arising from differences in sample sizes. Such variations in sample sizes can influence the detection and reporting of low-frequency markers like claudin-5 in mesothelioma, leading to differing conclusions in otherwise similar studies. Stefon et al. have reported that claudin-15 may be valuable in subtyping mesotheliomas (92). This finding is particularly significant from a prognostic standpoint, as different subtypes of mesothelioma are known to have varying implications for patient prognosis. The ability to accurately subtype mesotheliomas using markers like claudin-15 could therefore play a crucial role in predicting disease outcomes and tailoring treatment strategies to individual patient needs, enhancing the overall management of this challenging malignancy.

MPM and LUAC often present diagnostic challenges due to their overlapping histological features. Recent studies have highlighted the potential of claudin-4 as a differential marker in this context. The absence of claudin-4 has been found particularly useful in distinguishing between MPM and adenocarcinoma, as almost all carcinoma cases express claudin-4 (93). This contrast in claudin-4 expression between MPM and LUAC has been documented in various research articles (60, 94–98). These findings suggest the inclusion of claudin-4 level assessment could enhance the diagnostic accuracy when distinguishing between MPM and LUACs.

## 5 Therapeutic implications of claudins in lung cancer

The therapeutic potential of claudins in lung cancer has garnered increasing interest, particularly as the field moves towards more targeted approaches. Recent advancements have incorporated *Clostridium perfringens* enterotoxin (CPE), particularly due to its ability to bind to specific claudins overexpressed in cancer cells. Claudins such as claudins-3 and -4 serve as receptors for CPE, making it an ideal candidate for targetting claudins. This has led to the development of modified CPE variants, such as CPE-Mut3, which show enhanced binding to a broader range of claudins including CLDN-1 and CLDN-5. These modifications improve the targeting and cytotoxicity against claudin-overexpressing tumors, which is particularly beneficial for NSCLC patients where these claudins are prevalent (99). Furthermore, studies have shown that CPE-mediated therapies can disrupt tight junctions in cancer cells, leading to their destruction and offering a potential pathway for novel cancer treatments (100). Additionally, combining CPE with other therapeutic modalities, such as gold-nanoparticle-mediated laser intervention, has demonstrated significant reduction in tumor cell viability, further enhancing the therapeutic landscape (101). However, the success of these therapies depends on the accessibility of claudins on the cancer cell surface and the specific conditions under which CPE can exert its cytotoxic effects (102).

Beyond CPE, other therapeutic strategies targeting claudins have also shown promise. A groundbreaking study on CLDN6-specific CAR-T cells combined with an amplifying RNA vaccine has demonstrated promising results in patients with relapsed or refractory solid tumors, of which half had lung involvement. This approach leverages the specific expression of CLDN6 in tumors while avoiding on-target/off-tumor toxicity, a major challenge in CAR-T therapies for solid tumors. The ongoing Phase 1 BNT211-01 trial has reported a disease control rate of 67% and an objective response rate of 33%, with manageable safety profiles (103). This innovative therapy could open the door for new therapeutic opportunities; however, this would need to be tested in primary lung neoplasms.

In addition, histone deacetylase inhibitors like tricostatin A and quisinostat have been demonstrated to suppress claudin-2 expression, resulting in reduced tumor cell proliferation and migration, which could represent a potential therapeutic avenue for lung adenocarcinomas (104). Claudin-18, particularly its splice variant 2, has been identified as a viable target for therapeutic antibodies, although clinical trials have yet to show efficacy in its lung-specific form (105). Moreover, claudin-7 has been linked to increased cisplatin sensitivity by promoting pro-apoptotic pathways (106). However, the role of claudin-1 in drug resistance remains complex, with conflicting evidence regarding its impact on chemosensitivity (107, 108).

Conclusively, while claudin-targeting therapies, particularly those involving CPE, hold significant potential, ongoing research and clinical trials are essential to refine these approaches and fully realize their potential in lung cancer treatment.

## 6 Conclusion

In summary, our review has elucidated the complex role of claudins in the pathophysiology of lung cancer. These tight junction proteins are not only pivotal in maintaining cell polarity and barrier integrity but also significantly influence lung cancer progression and metastasis. The differential expression of claudins among various lung cancer subtypes offers insights into their potential as biomarkers for diagnosis and prognosis. Notably, the overexpression of certain claudins like claudin-2 and claudin-4 has been linked with increased malignancy and poor patient outcomes, while others such as claudin-1 show variable associations depending on the cancer subtype. Furthermore, the interaction of claudins with inflammatory pathways and their modulation by cytokines highlights their role in cancer microenvironment dynamics. The therapeutic potential of targeting specific claudins, supported by emerging studies, suggests a promising avenue for developing personalized treatment strategies, particularly in resistant cases. As research continues to unravel the multifaceted functions of claudins, it is imperative that future clinical trials are designed to explore and validate claudin-targeting therapies in lung cancer management.
